# Integrative Monitoring of Marine and Freshwater Harmful Algae in Washington State for Public Health Protection

**DOI:** 10.3390/toxins7041206

**Published:** 2015-04-09

**Authors:** Vera L. Trainer, F. Joan Hardy

**Affiliations:** 1 NOAA, Northwest Fisheries Science Center, Marine Biotoxins Program, Seattle, WA 98112, USA; 2 Washington State Department of Health, Olympia, WA 98504, USA; E-Mail: joan.hardy@doh.wa.gov

**Keywords:** domoic acid amnesic shellfish poisoning, saxitoxin, paralytic shellfish poisoning, okadaic acid, diarrhetic shellfish poisoning, freshwater HABs, anatoxin-a, microcystins, ORHAB, SoundToxins

## Abstract

The more frequent occurrence of both marine and freshwater toxic algal blooms and recent problems with new toxic events have increased the risk for illness and negatively impacted sustainable public access to safe shellfish and recreational waters in Washington State. Marine toxins that affect safe shellfish harvest in the state are the saxitoxins that cause paralytic shellfish poisoning (PSP), domoic acid that causes amnesic shellfish poisoning (ASP) and the first ever US closure in 2011 due to diarrhetic shellfish toxins that cause diarrhetic shellfish poisoning (DSP). Likewise, the freshwater toxins microcystins, anatoxin-a, cylindrospermopsins, and saxitoxins have been measured in state lakes, although cylindrospermopsins have not yet been measured above state regulatory guidance levels. This increased incidence of harmful algal blooms (HABs) has necessitated the partnering of state regulatory programs with citizen and user-fee sponsored monitoring efforts such as SoundToxins, the Olympic Region Harmful Algal Bloom (ORHAB) partnership and the state’s freshwater harmful algal bloom passive (opportunistic) surveillance program that allow citizens to share their observations with scientists. Through such integrated programs that provide an effective interface between formalized state and federal programs and observations by the general public, county staff and trained citizen volunteers, the best possible early warning systems can be instituted for surveillance of known HABs, as well as for the reporting and diagnosis of unusual events that may impact the future health of oceans, lakes, wildlife, and humans.

## 1. Introduction

Both marine and freshwater toxic algal blooms are believed to be occurring more frequently in lakes, estuaries and oceans of the U.S. Recent problems with new toxic events have increased the risk for illness and negatively impacted sustainable public access to safe shellfish and recreational waters in Washington State. To address these increasing threats to public health, monitoring programs have been strengthened through collaborations that include observations and analyses performed by local, state, and federal scientists, as well as volunteer groups.

Washington State produces the highest amount of commercially harvested mussels, clams and oysters in the nation with an estimated annual production of 39 thousand metric tons that generates over $77 million in sales [2]. This commercial harvest, together with recreational shellfish harvest on Washington’s public beaches by approximately 300,000 people, necessitates an effective and comprehensive monitoring program for biotoxins that can affect shellfish safety. If harmful algae producing natural toxins are present, toxins can collect in shellfish tissue and cause illness or even death in marine wildlife or people. The expansion of Washington’s shellfish growing areas is evidenced by the net gain of 27,811 acres approved for commercial shellfish production from 1991 to 2010 and an increase in beaches open for recreational harvesting from 78 in 2005 to 201 in 2010 [3,4]. However, marine toxins continue to pose a severe threat to shellfish safety. Closures due to paralytic shellfish toxins are annual occurrences. For example, in 2012, 453 shellfish tissue samples had concentrations of PSP above the regulatory level of 80 μg/100 g and 50 samples had concentrations above 1000 μg/100 g, resulting in closures of numerous commercial shellfishing areas to harvesting [4]. New toxic events are also entering the scene. In 2011, Washington had a confirmed case of diarrhetic shellfish poisoning (DSP), the first known illness from this marine biotoxin-related syndrome in the United States [5].

Toxic cyanobacteria have been observed in over 132 lakes in Washington State, resulting in animal and human illnesses and animal deaths in some lakes [6,7,8]. Toxic cyanobacteria and blooms occur in natural lakes, manmade reservoirs, and ponds, but especially those that are influenced by watershed development and pollution. Lakes that produce toxic blooms often provide citizens with vital recreational opportunities in addition to supplying drinking water. Closures of lakes due to toxic blooms have had economic impacts in lakes from all regions of the state resulting in closure of recreational areas and restriction of fishing. With the potential for cyanotoxins to bioaccumulate in fish, public health officials are concerned about exposure through consumption [9], and freshwater toxins from lake blooms have been observed downstream in marine shellfish [10]. Furthermore, in 2014, a lake in the Puget Sound lowlands that provides drinking water to over 500 households had its first toxic bloom, provoking intense scrutiny and public concern [8]. Regional or short-term monitoring programs and opportunistic surveillance indicate that toxic blooms are becoming more frequent in the state, potentially impacting public health, regional economies, and lifestyles of citizens who use the lakes.

**Table 1:** Regulated marine and freshwater toxins in Washington State. ^a^ Relative abundance values are used by SoundToxins and ORHAB partnerships to provide rapid, early warning of shellfish toxicity in the marine environment; ^b^ Cell count action level is >50,000 cells/L (large *Pseudo-nitzschia*) and >1,000,000 cells/L (small *Pseudo-nitzschia*); ^c^ Relative abundance values. For *Dinophysis*, cell count action level is >20,000 cells/L (“common”) or an increase from “present” to “common”; ELISA = enzyme-linked immunosorbent assay; LC/MS-MS = liquid chromatography tandem spectrometry; HPLC = high performance liquid chromatography; n/a = not applicable; nd = not done.

Toxins	Known causative organism(s) in WA	Regulatory Method	Action Level (Regulatory or Guidance)			Year of first known illness in WA State
			Shellfish	Water or particulate toxin	Relative cell abundance ^a^	
**Freshwater Toxins**						
Microcystins	*Microcystis*, *Anabaena*	ELISA	n/a	6 μg/L	nd	1976
	*Planktothrix*					
	*Aphanizomenon*					
	*Hapalosiphon*, *Nostoc*					
	*Anabaenopsis*					
	*Hapalosiphon*					
	*Gloeotrichia*					
Anatoxin-a	*Anabaena*	LC/MS-MS	n/a	1 μg/L	nd	1989
	*Aphanizomenon*					
	*Planktothrix*					
	*Oscillatoria*					
	*Cylindrospermopsis*					
	*Raphidiopsis*					
Cylindrospermopsin	*Aphanizomenon*	ELISA	n/a	4.5 μg/L	nd	n/a
	*Cylindrospermopsis*					
Saxitoxin	*Anabaena*	ELISA	n/a	75 μg/L	nd	n/a
	*Aphanizomenon*					
	*Planktothrix*					
	*Cylindrospermopsin*					
**Marine Toxins**						
Saxitoxins	*Alexandrium*	Mouse bioassay	80 μg/100 g	~100–200 ng/L STX equiv./L [11]	present	1942
Domoic acid	*Pseudo-nitzschia*	HPLC	20 ppm	~200 ng/L [12]	common or bloom ^b^	1991
Diarrhetic shellfish toxins	*Dinophysis*	LC/MS-MS	16 μg/g	~20 ng/L [13]	increase from present to common ^c^	2011

Here we provide an overview of marine and freshwater toxins in the region that endanger human health (Table 1) and describe the emergence of integrated, interagency monitoring programs for marine and freshwater toxins in Washington State, necessitated by increases in toxin threats to our valued shellfish and freshwater resources. First, we describe marine toxins, their algal hosts, biochemical activity, and historical trends in shellfish toxicity and illness events, followed by similar summary sections for the freshwater toxins. Finally, we provide recommendations for the future and suggestions for tools that can be used for integrative monitoring of biotoxins, marine and fresh water alike.

## 2. Shellfish Monitoring for Marine Toxins

Monitoring of shellfish safety is a critical function of the Office of Shellfish and Water Protection of the Washington State Department of Health (DOH). The shellfish toxicity surveillance program was initiated by the DOH in the early 1930s as a collaboration between DOH and the George Williams Hooper Foundation for Medical Research in San Francisco [14]. Initial monitoring by DOH focused on commercial shellfish and included recreational shellfish for the first time in the early 1990s. Since the 1930s, the DOH has measured biotoxins in shellfish from hundreds of locations in western Washington waterways in order to protect consumers from shellfish poisoning. When harmful levels of biotoxins are measured, alerts are issued by DOH to shellfish growers and harvesters, local health agencies, and tribes by newspaper, television, the DOH Biotoxin Hotline (1.800.562.5632), and the internet [15]. A highly structured Sentinel Monitoring Program was established in 1990 [16] to provide early warning of the onset of biotoxin concentrations in shellfish.

Through this Sentinel Monitoring Program, caged mussels are sampled at about 40 locations in Washington’s marine waters every 2 weeks thoughout the year. Generally the blue mussel, *Mytilus edulis*, is sampled; however, *M. galloprovincialis* and *M. californianus* are collected at a few Puget Sound sites. Wire mesh cages are stocked with mussels and suspended from floats and docks. Caged mussels sit for at least 1 week before they are sampled and are replenished as needed. At a few sites, natural-set mussels are harvested. Seventy to 100 mussels provide the 100 grams of tissue needed for analysis. Mussels are sealed into plastic bags, chilled with frozen gel packs, and shipped to the DOH laboratory in Seattle for analysis by mouse bioassay [17] (Table 1).

When toxins are detected above the regulatory level in shellfish, the harvest area is closed. It takes two shellfish samples of the same species from same area collected 7–10 days apart with acceptable levels of toxin to reopen a closed area to harvest. When the closure is in a commercial harvest area, all licensed shellfish companies in that area are notified to stop harvesting immediately. Commercial product that came from a closed area may also be recalled from the market.

## 3. Marine Toxins Affecting Public Health in Washington

### 3.1. Saxitoxins

#### 3.1.1. Activity and Source of Saxitoxins

Saxitoxins are among the most potent natural toxins known [18] that act by blocking sodium channels of nerves, impairing normal signal transmission [19,20]. More than 30 different saxitoxin analogues have been identified, including pure saxitoxin (STX), neosaxitoxin (neoSTX), the gonyautoxins (GTX) and decarbamoylsaxitoxin (dc-STX) of which STX, NeoSTX, GTX1 and dc-STX are the most toxic isomers. The term saxitoxin often refers to the entire suite of related neurotoxins produced by cyanobacteria and marine algae.

This suite of closely related tetrahydropurines (saxitoxins-STX) is also described as a group of carbamate alkaloid toxins that are either nonsulfated (STXs), singly sulfated (gonyautoxins, GTX), or doubly sulfated (C-toxins) [21]. Chemically, saxitoxin is stable and readily soluble in water, although it can be inactivated by treatment with a strong alkali. The half-lives for breakdown of a range of different saxitoxins in natural water have been shown to vary from 9 to 28 days, and gonyautoxins may persist in the environment for more than 3 months [22]. The toxicological database for STX-group toxins is limited and is comprised primarily of studies on acute toxicity following intraperitoneal (i.p.) administration. For monitoring purposes, toxicity equivalency factors (TEFs) have been applied to express the detected analogues (using high performance liquid chromatography, HPLC) in freshwater systems and the mouse bioassay for shellfish in marine systems) as STX equivalents (STX-equiv.). The Scientific Panel on Contaminants in the Food Chain (EFSA 2009) proposes the following TEFs based on acute intraperitoneal toxicity in mice: STX = 1, NeoSTX = 1, GTX1 = 1, GTX2 = 0.4, GTX3 = 0.6, GTX4 = 0.7, GTX5 = 0.1, GTX6 = 0.1, C2 = 0.1, C4 = 0.1, dc-STX = 1, dc-NeoSTX = 0.4, dc GTX2 = 0.2, GTX3 = 0.4, and 11-hydroxy-STX = 0.3.

The dinoflagellate *Alexandrium catenella* (Balech), previously described as belonging to the genus *Gonyaulax* (Whedon and Kofoid) or *Protogonyaulax* (Taylor), has been identified as the primary causative species of paralytic shellfish poisoning on the west coast of North America [23]. However, the name *A. fundyense* [24,25] has recently been proposed to replace all Group I strains of the *A. tamarense* species complex which includes the Washington *Alexandrium* isolates.

#### 3.1.2. Illness and Symptoms

Saxitoxins are toxic by ingestion and by inhalation, with inhalation leading to rapid respiratory collapse and death. Intoxication with saxitoxin can be a severe, life-threatening illness requiring immediate medical care. Most information on saxitoxin symptoms comes from exposure through consumption of shellfish. Within minutes of eating toxic shellfish, a person would initially develop tingling of the lips and tongue. However, it can take up to an hour or two to develop tingling, depending on the dose and individual tolerance, followed by numbness and weakness with loss of control of arms and legs, developing into difficulty in breathing. Some people feel nauseated or experience a sense of floating after saxitoxin exposure. If a person consumes enough saxitoxin, muscles of the chest and abdomen become paralyzed, including muscles used for breathing, and the victim can suffocate. Terminal stages of saxitoxin poisoning can occur 2–12 h after exposure, and death from PSP has occurred in less than 30 min [26].

Diagnosis of saxitoxin poisoning is confirmed by detection of toxin in the food, water, stomach contents, or environmental samples. Artificial respiration is used to support breathing; when such support is applied within 12 h of exposure, recovery usually is complete with no lasting side effects [27,28,29]. Stomach evacuation can be conducted if exposure is through ingestion. No antidote against saxitoxin exposure has been developed for human use.

#### 3.1.3. Washington Occurrences

**Figure 1:**
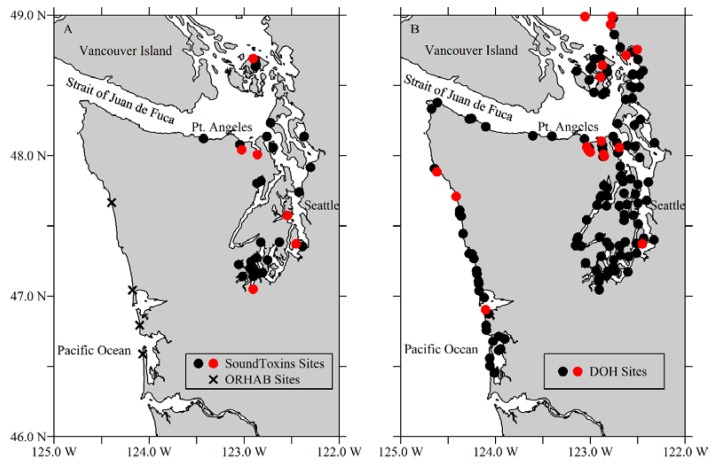
Phytoplankton monitoring locations and DOH shellfish biotoxin management sites in 2014. A. SoundToxins monitoring sites (black circles), including locations where *Dinophysis* was quantified as common or greater (red circles), B. DOH shellfish biotoxin monitoring sites (black circles) include locations where diarrhetic shellfish toxins were at or above the regulatory level of 16 μg/kg (red circles). Routinely monitored, core ORHAB sites are marked with X.

Closures of recreational shellfish harvesting due to paralytic shellfish toxins (PSTs) have been imposed in Washington State since 1942 when three Native American fatalities occurred in the town of Sekiu on the Strait of Juan de Fuca [13]. At that time, the Washington Department of Fisheries imposed annual closures for all shellfish harvest except razor clams from 1 April to 31 October in the area west of Dungeness Spit (near Port Angeles, WA, Figure 1) including the Pacific coast to the Columbia River [30]. The shellfish surveillance program for PSTs was temporarily stopped in 1946 when it was believed that the seasonal blanket closure was adequately protecting public health. However, an outbreak of PSP on eastern Vancouver Island in 1957 [31] resulted in a mandatory monitoring program for PSTs in all commercial shellfish in Washington. Illnesses due to PSP were not reported in Puget Sound prior to 1978, but widespread toxicity occurred that year throughout much of the central basin [30]. High numbers of illnesses include 14 in 1978, nine in 2000, and 7 in 2012, all in Puget Sound [32,33,34] (Table 2). Toxins causing PSP are now found in most areas of Puget Sound after a massive event in 1978 that caused spreading into the main basin, then further migration into the southernmost reaches of the Sound in the 1980s and 1990s. Multiple closures due to PSTs occur annually at many locations throughout Puget Sound.

**Table 2:** Marine biotoxin-related illnesses, 1942–2014. ^a^ Includes 3 deaths; ^b^ Probable illnesses with only one illness confirmed; ^c^ Domoic acid concentration in ppm. Source: DOH.

Year	Month	Illnesses	Shellfish	Toxin Concentration (μg/100 g)
1942	May	6 PSP ^a^	Mussels, Clams	3500
1978	August	4 PSP	Scallops	2597
1978	September	10 PSP	Mussels	1415
1979	July	3 PSP	Clams	2597
1985	September	2 PSP	Scallops	1107
1985	November	1 PSP	Scallops	338
1988	May	2 PSP	Clams	1200
1988	September	5 PSP	Oysters	2171
1991	October	25 ASP ^b^	Razor Clams	26 ppm ^c^
1998	October	5 PSP	Mussels	10,928
2000	August	9 PSP	Mussels	13,769
2007	April	1 PSP	Clams	709
2011	June	3 DSP	Mussels	160
2012	August	1 PSP	Mussels	1621
2012	September	1 PSP	Mussels	10,304
2012	September	7 PSP	Mussels	6250

### 3.2. Domoic Acid

#### 3.2.1. Activity and Source of Domoic Acid

Several species of pennate, chain-forming diatoms in the genus *Pseudo-nitzschia* are known to produce domoic acid (DA), a toxin that bioaccumulates through the food chain to shellfish and planktivorous fish, then to vertebrates such as birds, marine mammals, and humans. The toxin, DA, acts at the same nerve receptor as glutamate, the major excitatory neurotransmitter in the mammalian central nervous system that is responsible for many of the functions within the brain, including learning and memory. Several comprehensive recent reviews are available for more information on *Pseudo-nitzschia* and DA [35,36,37,38,39].

#### 3.2.2. Illness and Symptoms

Domoic acid poisoning is formally known as amnesic shellfish poisoning in humans. Gastrointestinal symptoms can appear 24 h after ingestion of shellfish containing DA and may include vomiting, nausea, diarrhea, abdominal cramps and bleeding in the gastrointestinal system. Neurological symptoms in more severe cases can take hours to three days to appear and include headaches, hallucinations, confusion and impairment of short-term memory, unstable blood pressure, cardiac arrhythmia and coma [40]. People poisoned with very high doses of the toxin or those who display risk factors such as old age or renal failure can die after exposure.

#### 3.2.3. Washington Occurrences

The razor clam and Dungeness crab fisheries on the outer coast of Washington have been plagued by DA closures since 1991 [41,42,43]. Commercial, recreational and subsistence razor clam fisheries suffered total coastwide closures in 1991, 1998 and 2002. However, due to enhanced information about specific locations of *Pseudo-nitzschia* species attributable to the monitoring efforts of the ORHAB partnership, formed in 2000, selective closures were possible in 2001 and 2003–2005. Because razor clams can retain DA for periods of up to a year due to the presence of a high affinity glutamate binding protein [44], closures on the outer coast lasting for up to a year caused serious economic hardship to the tribal communities which rely on this subsistence fishery.

DA closures occurred in Puget Sound in 2003 and 2005, causing great concern to shellfish managers. To date, concentrations of DA below the regulatory level of 20 ppm have been detected in Puget Sound blue mussel (*Mytilus edulis*), littleneck clam (*Protothaca staminea*), geoduck clam (*Panopea abrupta*), manila clam (*Tapes philippinarum*), Pacific oyster (*Crassostrea gigas*), and Dungeness crab (*Cancer magister*) [45]. If future DA concentrations are found at levels in excess of the regulatory level in more areas of Puget Sound, resulting economic losses could be severe.

### 3.3. Diarrhetic Shellfish Toxins

#### 3.3.1. Activity of Diarrhetic Shellfish Toxins

These lipophilic toxins that are often found in combination in shellfish can be divided into four groups with different chemical structures and relative toxicities in humans: Okadaic acid (OA and its derivatives, the DTXs; the pectenotoxins (PTXs); the yessotoxins (YTXs); and the azaspiracids (AZAs). Both OA and the DTXs are lipid polyethers with inhibitory effects on protein phosphatases [46,47] and are the only toxins of the DSP group that can cause diarrhea in mammals [48]. The PTXs and YTXs are toxic in animal studies [49] but have not yet been associated with human poisonings [50]. The AZAs were first described after several people became ill after consuming contaminated mussels in Ireland [51] and have recently been measured in shellfish from Washington State at low concentrations [5].

#### 3.3.2. Illness and Symptoms

Diarrhetic shellfish poisoning (DSP) is a human syndrome caused by consumption of shellfish contaminated by toxins produced by *Dinophysi*s and benthic species of *Prorocentrum* [52,53,54]. However, no DSP outbreaks associated with *Prorocentrum* have been described in Washington. DSP symptoms are gastrointestinal and include diarrhea, nausea, vomiting, and abdominal distress starting a few minutes to hours after ingestion of the toxic shellfish. Recovery occurs within three days [55].

#### 3.3.3. Washington Occurrences

The first clinical report of DSP in the in the Pacific Northwest and in the U.S. with coincident high concentrations of diarrhetic shellfish toxins was due to the consumption of toxin-laced mussels collected from a pier at Sequim Bay State Park in northwest Washington in June 2011. Nine mussel samples collected immediately after the illnesses were reported, contained toxins at 2–10 times above the regulatory level. Coincidently, about 60 DSP illnesses associated with the ingestion of mussels occurred on Salt Spring Island, British Columbia, the first reports of DSP in western Canada [56], resulting in the recall of almost 14,000 kg of shellfish. Sites with shellfish testing positive for diarrhetic shellfish toxins above the regulatory level of 16 μg/g in 2014 are shown in Figure 1.

## 4. Integrative Monitoring of Marine Toxins in Washington

In most coastal regions of the world, shellfish harvesting closures based on monitoring for toxins are primarily reactionary. These systems have succeeded in protecting human health but often have led to conservative, blanket closures of shellfish harvesting operations, thereby negatively impacting the economy of the shellfish industry. The recent appearance of new toxins in Washington challenges the capacity and effectiveness of monitoring programs that are based solely on assessment of shellfish toxicity. The National Shellfish Sanitation Program (NSSP) recommends phytoplankton monitoring as an early warning for the control of shellfish safety to provide the assurance that states are taking adequate measures to prevent harvesting, shipping, and consumption of toxic shellfish. The plan encourages communication with other states, researchers and other environmental professionals [57,58,59]. Washington is one of the US states that has successfully integrated phytoplankton and shellfish monitoring through collaboration of DOH with two phytoplankton monitoring programs, the ORHAB partnership on the Pacific coast of Washington [12] and the SoundToxins program in Puget Sound [60].

The ORHAB partnership was established in 1999 and uses a combination of analytical techniques, including weekly quantification of total numbers of harmful algae using microscopes and determination of DA concentration in seawater and razor clams, to give an effective early warning of shellfish toxin events (see Figure 1 for primary ORHAB sites denoted with X). Because razor clams are the main recreationally-harvested shellfish on the outer coast of Washington and accumulate and retain more DA than any other shellfish [44], the ORHAB early warning system is focused solely on DA testing in these shellfish using enzyme-linked immunosorbent assay (ELISA). The efficacy and accuracy of ELISA for diarrhetic shellfish toxin screening are currently being tested for eventual use by the State’s phytoplankton monitoring programs [61].

Using ORHAB as a model, SoundToxins was established in 2006 and has grown from four partner sites in 2006 to >30 monitoring locations today (Figure 1). Seawater samples are collected weekly by the participants at ORHAB sites on the outer coast and SoundToxins sites throughout Puget Sound and are analyzed for salinity, temperature, nutrients, chlorophyll, and particulate toxins, including paralytic shellfish toxins, DA, and diarrhetic shellfish toxins. Phytoplankton relative abundance focuses on four target genera *Pseudo-nitzschia*, *Alexandrium*, *Dinophysis* species, and *Heterosigma akashiwo*. In addition, SoundToxins participants recently have assisted with the identification of *Azadinium* species in Puget Sound.

Through its weekly monitoring of phytoplankton at sites around Puget Sound, the SoundToxins partnership has allowed the state to target monitoring for diarrhetic shellfish toxins to those sites that have the greatest risk of toxicity due to increases in relative abundance of *Dinophysis* spp. from present to common or greater (Figure 1, see definitions in Table 1). SoundToxins participants, including environmental learning centers, Native Tribes, shellfish growers, state and federal researchers and private citizens enter weekly phytoplankton relative abundances into a web-based system [60], allowing rapid visualization of data and decision making by DOH officials. Future improvements will include closer pairing of SoundToxins phytoplankton monitoring sites with shellfish harvesting areas (Figure 1) and rapid toxin testing at the sites of shellfish harvest by volunteers to provide a swift assessment of toxin risk for managers.

## 5. Monitoring for Freshwater Cyanobacteria and Their Toxins

Cyanobacteria blooms are common in numerous Washington lakes. Cyanobacteria (also known as blue-green algae) can create toxins collectively called cyanotoxins. A documented public health concern, cyanotoxins include the liver toxins microcystins and cylindrospermopsins and the nerve toxins anatoxin-a and saxitoxins. Historically, many animals have become ill or have died after exposure to cyanotoxins in state lakes. To address this issue, the DOH and Washington State Department of Ecology (Ecology) have conducted surveillance of blooms and human and animal illnesses related to cyanotoxin exposure for several years.

Freshwater algae and cyanobacteria produce blooms that may be non-toxic one day but may become toxic the next day or later in the growing season. The only way to know whether a cyanobacterial bloom is toxic is to test for the presence of toxins. Due in part to citizens’ mounting concerns over potential health impacts from exposure to rapidly appearing freshwater cyanotoxins, the state legislature created and funded a Freshwater Algae Control Program in 2005. This Ecology program provides funds to conduct toxicity testing by King County Environmental Laboratory (KCEL) on samples collected by local health jurisdictions, lake managers, other agencies or lake residents from lakes with blooms. Originally, analysis was done on samples collected under the passive surveillance program only for microcystins, but KCEL later developed the capacity to test for anatoxin-a, cylindrospermopsins, and saxitoxins.

During initial development of the Freshwater Algae Control Program, stakeholders requested that state guidelines be developed to help with interpretation of toxicity results. In the absence of recreational guidance (based on actual toxicity levels and not cell concentrations) from the United States or the World Health Organization (WHO) for microcystins and anatoxin-a, DOH developed provisional guidance values (health-based recommendations that are not formal regulatory values) for both cyanotoxins based on a review of toxicology literature and standard risk assessment methods [62]. Later, DOH developed provisional recreational guidance for saxitoxins and cylindrospermopsins [63]. As part of the effort to provide assistance to local health jurisdictions and lake managers, DOH also developed a lake protocol that incorporated these guidance values as a reference for use by managers, agencies, and local health jurisdictions (LHJs) (Table 1). While the most likely exposure pathways to freshwater cyanotoxins are through recreational contact or contaminated drinking water, long-term chronic ingestion via drinking water and exposure through consumption of fish and shellfish were not considered in development of recreational guidance. Recreational exposure includes activities such as swimming, wind surfing, jet skiing, and water skiing. The calculations used to determine these provisional recreational guidance values are described below.

DOH incorporated the approach used by Oregon and Vermont in initial derivation of recreational guidance for microcystins [64]. Oregon has recently updated its guidance values to include anatoxin-a, cylindrospermopsin, and saxitoxin and to address acute or short-term exposures for human drinking water exposure, human recreational exposure, and dog-specific exposures [65]. DOH calculations assume a default child’s body weight (BW) of 15 kg and an ingestion rate (IR) of 0.1 L based on 2 h exposure by a swimmer or other lake user with an exposure lasting for two hours per day [62]. Using the WHO tolerable daily intake (TDI) of 0.04 μg/kg-day [66] and other assumptions, above, DOH recommends a provisional recreational guidance value of 6 μg/L for microcystins, calculated as follows:

(1) $$\text{Recreational guidance value }\left(\text{ µg}/\text{L}\right)=\frac{\text{TDI }\times \text{BW}}{\text{IR}}$$

DOH recommends a provisional recreational guidance value of 4.5 µg/L cylindrospermopsin, assuming a subchronic RfD of 0.03 µg/kg-day (EPA 2006) calculated using EPA assumptions, as above (RfD, in place of a TDI in the above equation). Similarly, for saxitoxins, DOH recommends a provisional recreational guidance value of 75 µg/L saxitoxin, calculated using an acute RfD developed by the European Food Safety Association [67] based on acute toxicity of STX-equivalent intoxications in humans (>500 individuals) [63]. For anatoxin-a, DOH recommends a provisional recreational guidance value of 1 µg/L based on a systemic toxicity study in mice exposed to anatoxin-a for 28 days [62,68]. When an acute reference dose (RfD) or estimate of daily oral exposure becomes available for anatoxin-a, DOH will reassess this interim anatoxin-a guidance value. All recommended recreational guidance values are considered “provisional” and will be reassessed when national or international guidance values become available.

**Figure 2:**
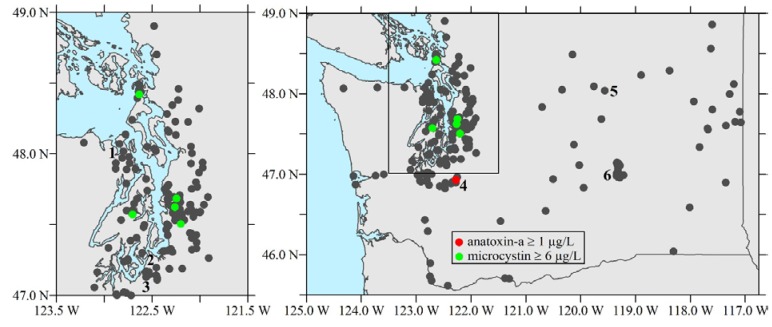
Washington lakes sampled for freshwater biotoxins (shown here are January 2014 data from [8]). Site names mentioned in the text are numbered: 1. Anderson Lake, 2. Waughop Lake, 3. American Lake, 4. Clear Lake, 5. Rufus Woods Lake, 6. Potholes Reservoir. Due to the size of the figure, sampled lakes are not visible.

In 2009, DOH and partners began a five-year cooperative agreement with the Centers for Disease Control and Prevention (CDC) to expand efforts to address HABs in Washington. Part of this project involved monitoring 30 Puget Sound lowland lakes for microcystins and anatoxin-a, and by 2010 the project added cylindrospermopsin and saxitoxin (Figure 2). Of the four cyanotoxins, microcystins were most frequently observed in the region, followed by anatoxin-a. Cylindrospermopsin and saxitoxin were each observed in only two Puget Sound lakes during the 2009 and 2010 sampling seasons of the 30-lake monitoring project.

## 6. Summary of Freshwater Toxins Affecting Public Health in Washington

Early in the program, DOH identified a list of cyanobacteria genera and species of concern for lakes in Washington. Toxicity testing is recommended when lake samples contain the following genera: *Microcystis*, *Anabaena*, *Aphanizomenon*, *Gloeotrichia*, *Oscillatoria/Planktothrix*, *Cylindrospermopsis*, *Lyngbya*, and/or *Nostoc.* In a summary report for the 2008–2009 state legislature, the top three toxic cyanobacteria genera in Washington lakes were identified as *Anabaena*, *Aphanizomenon*, and *Microcystis* [69]. *Gloeotrichia* was also included because a recent study confirmed microcystin-LR production by *Gloeotrichia echinulata* [70], and exposure to this genus has led to reports of human health impacts in Washington lakes.

Cyanotoxins are a diverse group of natural toxins that fall into three broad chemical structure groups [66,71]. These are cyclic peptides (microcystins and nodularin), alkaloids (anatoxins, saxitoxins, cylindrospermopsin, aplysiatoxins, and lyngbyatoxin), and lipopolysaccharides (irritants). Anatoxin-a(s) is a naturally-occurring organophosphate. Some genera, especially *Anabaena*, can produce both neuro- and hepatotoxins. If a toxic algal bloom contains both types of toxins, signs of neurotoxicity are usually observed first. Neurotoxic effects occur within minutes whereas effects due to liver toxins take one to a few hours to appear. Below we describe the freshwater toxins, microcystins, anatoxin-a, cylindrospermopsins and saxitoxins, which currently are monitored in Washington lakes.

### 6.1. Microcystins

#### 6.1.1. Microcystins

Microcystins are the most thoroughly investigated cyanobacterial toxins [72]. At least 90 structural variants have been identified, and microcystin-LR is the variant most commonly found in cyanobacteria [73,74,75]. Microcystins have been identified in *Anabaena*, *Microcystis*, *Oscillatoria* (*Planktothrix*), *Nostoc* and *Anabaenopsis* species and from the terrestrial genus *Hapalosiphon* [66]. More than one microcystin may be found in a particular cyanobacteria strain. Microcystins are cyclic heptapeptides that primarily affect the liver in animals. A lethal dose of microcystins in vertebrates causes death by liver necrosis within hours or up to a few days. Microcystins block protein phosphatases 1 and 2A (important molecular switches in all eukaryotic cells) using an irreversible covalent bond [76] in [77]. Liver injury is likely to go unnoticed and results in (external) noticeable symptoms only when it is severe [77]. Other studies have shown that microcystin toxicity is cumulative [78]. Researchers suspect microcystins are liver carcinogens, which could increase cancer risk to humans following continuous, low level exposure.

#### 6.1.2. Illness and Symptoms

Symptoms of microcystin poisoning may take 30 min to 24 h to appear, depending upon the size of the animal affected and the amount of toxic bloom consumed. Gross and histopathologic lesions caused by microcystins are quite similar among species, although species sensitivity and signs of poisoning can vary depending on the type of exposure. One of the earliest effects (15–30 min) of microcystin poisoning is increased serum concentration of bile acids, alkaline phosphatase, γ-glutamyltransferase, and aspartate aminotransferase. Microcystin symptoms in mammals and other animals may include jaundice, shock, abdominal pain and distention, weakness, nausea and vomiting, severe thirst, rapid and weak pulse, and death. It is likely that the number of incidents with low-level symptoms such as nausea, vomiting and diarrhea associated with recreational exposure to cyanobacterial toxins are underreported. Death may occur following exposure to very high concentrations within a few hours (usually within 4–24 h) or up to a few days. Death is due to intrahepatic hemorrhage and hypovolemic shock. In animals that survive more than a few hours, hyperkalemia or hypoglycemia, or both, may lead to death from liver failure within a few days [79]. Surviving animals have a good chance for recovery because the toxins have a steep dose-response curve. Activated charcoal oral slurry is likely to benefit exposed animals, even though therapies for cyanobacterial poisonings have not been investigated in detail.

#### 6.1.3. Washington Occurrences

**Figure 3:**
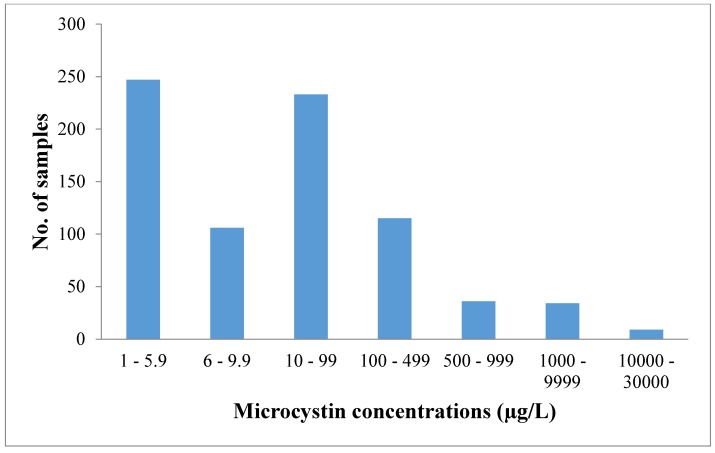
Distribution of the number of samples above 1 μg/L microcystin from 2009 through October 2014.

**Figure 4:**
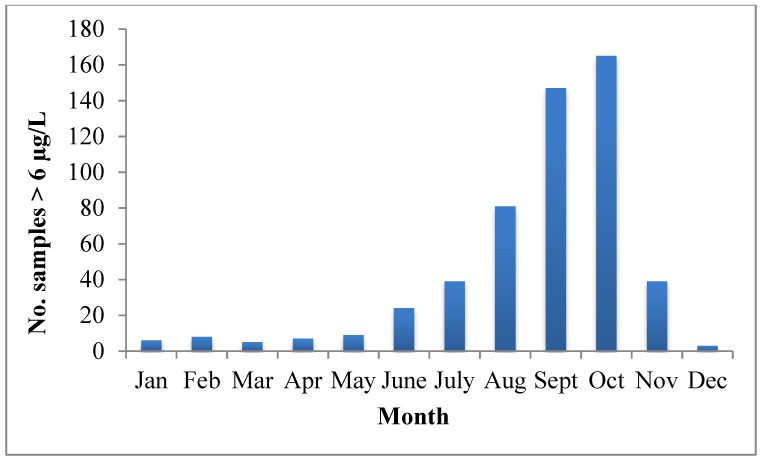
Monthly distribution of the sum of the number of microcystin samples above state recreational guidance value of 6 μg/L from 2009 through October 2014.

Microcystins are the most abundant cyanotoxins in Washington lakes. From the beginning of 2009 through the end of October 2014, 535 samples (representing 60 waterbodies) were observed with microcystin concentrations >6 µg/L (Figure 3). The number of samples above 6 µg/L each year ranged from 68 to 114 (2009–2013). The distribution of concentrations indicates that the majority of samples fell between 6 and 100 µg/L; 9 samples were above 10,000 µg/L microcystins (Figure 3). Seasonal distribution of microcystin samples with concentrations >6µg/L illustrates that the number of samples greater than the state’s recreational guidance value increases steadily from June through October (Figure 4).

### 6.2. Anatoxin-a

#### 6.2.1. Activity and Source of Anatoxin-a

Anatoxin-a is one of three neurotoxic alkaloids that have been isolated from cyanobacteria [72]. It is produced by various species of cyanobacteria including *Anabaena*, *Planktothrix* (*Oscillatoria*), *Aphanizomenon*, *Cylindrospermum* and *Microcystis* spp. Anatoxin-a was first detected in Canada in the 1960s [80]. Between 1961 and 1975, cattle and dog poisonings associated with *Anabaena flos-aquae* blooms occurred in six locations in Canada. Most anatoxin-a has been detected in Europe. Second to Europe, most reports of anatoxin-a have been in North America [74].

Anatoxin-a is a bicyclic secondary amine. It binds to the nicotinic acetylcholine receptor at the axon terminal at the neuromuscular interface [73,74]. Binding of anatoxin-a is irreversible causing the sodium channel to be locked in an open position, resulting in symptoms in humans including overstimulation, fatigue, and eventual paralysis. In the respiratory system, anatoxin-a exposure results in a lack of oxygen to the brain, subsequent convulsions and death by suffocation. Anatoxin-a is about 20 times more potent than acetylcholine, a compound involved in transmission of nerve impulses [74].

Alkaloid toxins are more likely to be present in free (non-cellular) form in water than the cyclic peptide toxins microcystins and nodularin [77]. While microcystins appear to be more common than freshwater neurotoxins, the latter have caused severe animal poisonings in North America, Europe and Australia [77]. Anatoxin-a degrades readily to nontoxic products upon exposure to sunlight and at a high pH [74]. In natural blooms in eutrophic lakes, the anatoxin-a half-life is typically less than 24 h, while its half-life in the laboratory is about five days [66]. This rapid degradation of anatoxin-a presents problems with determining accurate toxin concentrations associated with exposures. According to Botana [74], samples should be protected from light and acidified prior to storage at −20 °C in order to limit anatoxin-a degradation.

#### 6.2.2. Illness and Symptoms

Neurotoxins are notoriously rapid acting poisons; anatoxin-a was originally called very fast death factor (VFDF) due to its potency [74]. Animal illness and death may occur within a few minutes to a few hours after exposure, depending on the size of the animal and amount of toxic bloom consumed. An animal with anatoxin-a toxicosis may exhibit staggering, paralysis, muscle twitching, gasping, convulsions, backward arching of neck (in birds), and death. Livestock that drink large amounts of contaminated water and pets that lick scum on their fur are at highest risk from anatoxin-a exposure. While anatoxin-a is largely retained within cells when conditions for growth are favorable, toxins will be liberated in the gastrointestinal tract if water containing toxic cells is consumed [66,74]. However, ingestion of a sublethal dose of these neurotoxins leaves no chronic effects and recovery appears to be complete with no ongoing injury [77]. Exposure leaves no sign of organ damage and residual toxin is rapidly degraded [74].

The first report of an animal illness in Washington due to a freshwater toxic bloom occurred in 1976 in Spokane County [7]. Four dogs died after drinking water during a toxic *Anabaena* bloom and an additional seven dogs, one horse, and one cow were reportedly sickened [81]. In the 1980s, another two hunting dogs died in eastern Washington, and five cats died during a toxic *Anabaena* bloom in American Lake, Pierce County. More recently, two dogs died after exposure to a toxic *Anabaena* bloom in Anderson Lake (2006), and two hunting dogs died in the Potholes Reservoir after exposure to a toxic bloom (2007). Each year roughly 4–5 reports of animal illness (including cats, dogs, cows, elk, and horses) are investigated, with approximately 2 probable or confirmed cases per year. Outreach and education efforts such as posting signs at lakes with confirmed toxicity began in 2009 and are thought to have decreased pet exposures in lakes with blooms [82].

#### 6.2.3. Washington Occurrences

**Figure 5:**
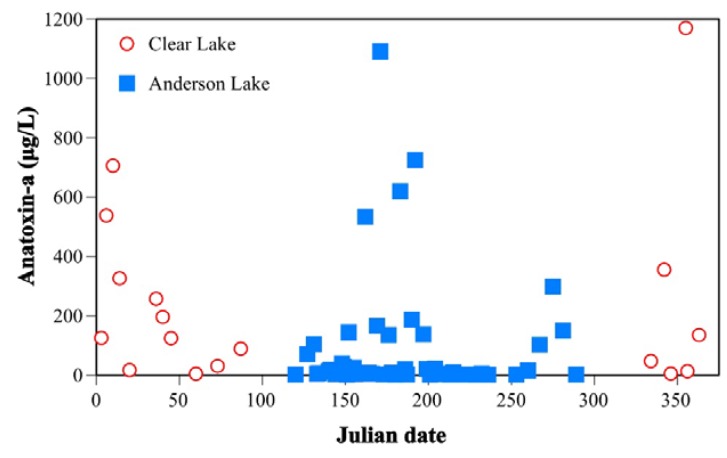
Seasonal distribution of anatoxin-a concentrations above 1 μg/L in Clear and Anderson Lakes.

Three state waterbodies have long-term reoccurring anatoxin-a blooms with unique seasonal patterns [8]. For example, Clear Lake, Pierce County, exhibited blooms three years in a row that became toxic in late fall and continued through the winter (maximum 1170 µg/L anatoxin-a). Testing of Anderson Lake, Jefferson County, from 2009 to 2014 showed reoccurring blooms that began in April, May, or June in most years and continued through August, September, or October (maximum 1090 µg/L anatoxin-a, June 2011; Figure 2 and Figure 5). Rufus Woods Lake, a reservoir behind Chief Joseph Dam on the Columbia River (for locations, see Figure 2), also has reoccurring blooms producing anatoxin-a with a unique seasonal pattern: July and August 2011; July, August, September 2012; May through September in 2013; and May through July in 2014 (maximum 110 µg/L anatoxin-a, July 2012).

Seasonal distribution of anatoxin-a concentrations above 1.0 µg/L was determined for 11 other state lakes and reservoirs. Levels above the state recreational guidance value were observed during each month of the year at various sites around the state. Ten lakes produced only one to three samples with anatoxin-a above 1.0 µg/L (maximum 592 µg/L). Most short-term blooms occurred in September, October, November or December.

### 6.3. Cylindrospermopsin

#### 6.3.1. Activity and Source of Cylindrospermopsin

Cylindrospermopsin is comprised of a tricyclic guanidine moiety combined with a hydroxymethyl uracil. Production of the toxin is strain- not species-specific [83]. Cylindrospermopsin exhibits a completely different mechanism of toxicity than the liver toxin microcystin [84,85,86]. Damage to cells is caused by blockage of key protein and enzyme functions, thereby inhibiting protein synthesis. Cylindrospermopsin targets the liver and kidneys but can also injure the lung, spleen, thymus, and heart as demonstrated in mouse studies [66,72,87,88]. Animal toxicity studies also suggest that cylindrospermopsin may be carcinogenic [72,89] and may produce genotoxicity in a human lymphoblastoid cell line [90]. Laboratory studies have shown that some of the compounds produced by *Cylindrospermopsis* may be carcinogenic and genotoxic [83,90,91,92,93]

Cylindrospermopsin is found in certain strains of five genera: *Cylindrospermopsis raciborskii* (Australia, Hungary, and the U.S.), *Umezakia natans* (Japan), *Anabaena bergii* and *Raphidiopsis curvata* [94], and *Aphanizomenon ovalisporum* (Australia, Israel) [95]. It is most commonly observed in tropical and subtropical waters of Australia [83]. The first report of animal poisonings attributed to cylindrospermopsin was in drinking water in a farm pond in Queensland, Australia, where it was responsible for cattle deaths [96]. Further, *Cylindrospermopsis raciborskii* was implicated in one of the most significant cases of human poisoning from exposure to a cyanobacterial toxin in 1979 on Palm Island, northern Queensland, Australia. Generally, toxins are retained in cyanobacterial cells when conditions are favorable; however studies have shown that it is not uncommon for 70%–98% of total cylindrospermopsin produced by cells to be dissolved in the water [83,97].

#### 6.3.2. Illness and Symptoms

Symptoms of exposure to cylindrospermopsin include nausea, vomiting, diarrhea, abdominal tenderness, pain, and acute liver failure. Clinical symptoms after exposure to cylindrospermopsin may not appear immediately but may occur several days later. Thus, it is often difficult to determine a cause-effect relationship between cylindrospermopsin exposure and symptoms.

The degree of the cyanotoxin impact for cylindrospermopsin and other cyanotoxins is influenced by animal size, species sensitivity, and individual sensitivity. According to the Merck Veterinary Manual, animals may need to ingest only a few ounces or up to several gallons to experience acute or lethal toxicity, depending on bloom densities and toxin content [79]. After removal from the contaminated water supply, affected animals should be placed in a protected area out of direct sunlight. The animal should have access to an unrestricted supply of clean water and good quality feed. Surviving animals have a good chance for recovery because both hepatotoxins and neurotoxins have a steep dose-response curve. Although no therapeutic antagonist has been found to be effective against cylindrospermopsin, activated charcoal oral slurry is likely to benefit exposed animals. An ion-exchange resin such as cholestyramine has proved useful to absorb the toxins from the gastrointestinal tract [79].

#### 6.3.3. Washington Occurrences

The state’s passive surveillance effort and monitoring results from the CDC 30-lake study show that cylindrospermopsins have been found in only six Washington lakes at very low concentrations. No results were above the state recreational guidance value of 4.5 µg/L cylindrospermopsins, with concentrations above the minimum detection level (MDL; 0.10 µg/L) ranging from 0.11 to 1.12 µg/L.

### 6.4. Saxitoxins

#### 6.4.1. Source of Freshwater Saxitoxins

Saxitoxins are found in both marine and freshwater systems and have been observed in numerous lakes around the world. Their toxicity has been described in detail, above. Cyanobacteria genera that are documented as producing saxitoxin include *Aphanizomenon sp*. (U.S.); *Aphanizomenon gracile*, *Aphanizomenon issatschenkoi*, *and Aphanizomenon flos-aqua* (Europe); *Anabaena circinalis* (Australia); *Anabaena lemmermannii* (Denmark); *Lyngbya wollei* (U.S.); *Cylindrospermopsis* (Brazil); and *Planktothrix* (Italy) [21,98,99,100].

#### 6.4.2. Washington Occurrences

Since 2009, saxitoxins have been detected in ten state lakes and one pond. Waughop Lake was the only waterbody with multiple samples higher than the MDL (0.020 µg/L), one of which was above the state recreational guidance value of 75 µg/L (193 µg/L, August 2009). Saxitoxin concentrations in the other lakes and pond ranged from 0.021 to 71.0 µg/L. With the exception of Waughop Lake (Figure 2), saxitoxins do not occur at levels of human health concern.

## 7. Washington Lakes: Three-Tiered Approach to Managing Lakes with Cyanobacterial Blooms

DOH recommends a three-tiered approach for managing toxic or potentially toxic cyanobacterial blooms. The approach applies recreational guidance values derived by DOH for managing Washington lakes (Table 1). Observers look for developing blooms and surface accumulations that can occur in any nutrient-rich water such as lakes, ponds, or river embayments. Upon notification of a potential bloom, the LHJ or other agency staff (or lake resident) will: (1) obtain a sample number from the state Freshwater Algae website [8], (2) sample the water body experiencing the bloom, then (3) send the sample to the laboratory for toxicity tests. Sampling and shipping directions are available at the website [8] or from Ecology’s Freshwater Algae Control Program [101].

At present the KCEL is under contract with Ecology to test for microcystins, anatoxin-a, cylindrospermopsin, and saxitoxin. Results of toxicity analyses are incorporated into the Freshwater Algae website as they are received from the laboratory. In Washington, local jurisdictions have the authority to post advisories on water bodies within their districts (RCW 70.05.070) and actions taken such as posting or closing a lake based on toxicity results are published on the website and on Ecology’s list serve.

Tier I

**Figure 6:**
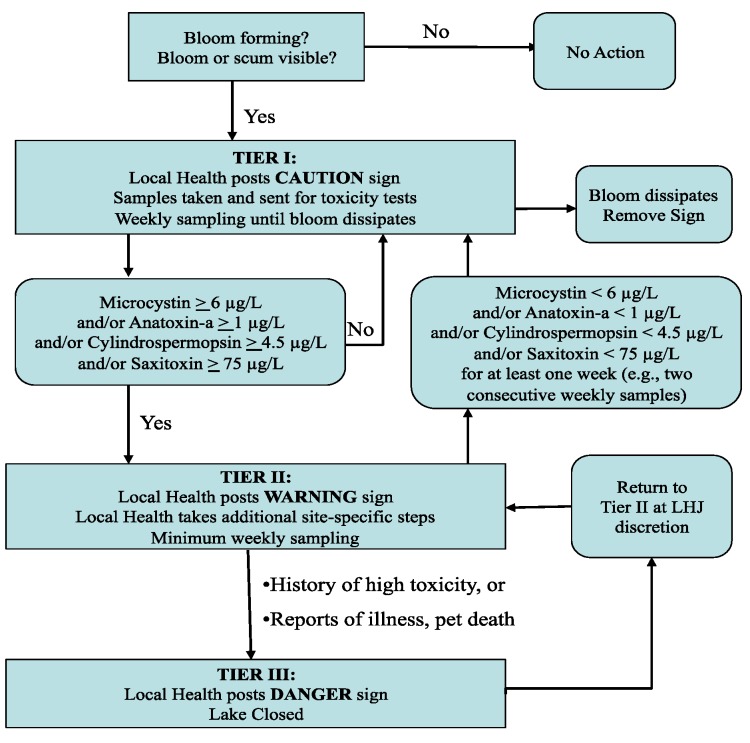
Three-tiered approach to managing Washington waterbodies experiencing cyanobacterial blooms.

**Figure 7:**
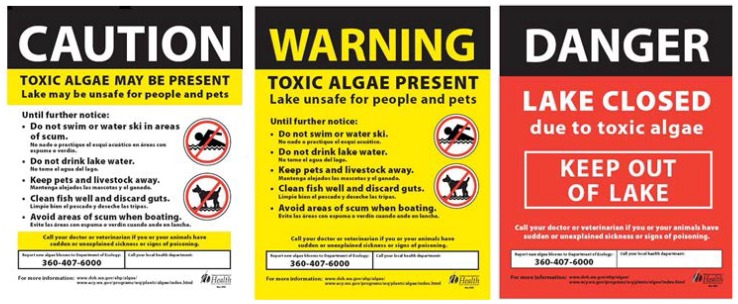
Advisory signs used in Washington’s Freshwater Algae Control Program.

A sample of a visible cyanobacteria bloom or scum is sent for phytoplankton examination and toxicity testing. If the sample is dominated by potentially toxic cyanobacteria, the LHJ should post a CAUTION sign (Figure 6 and Figure 7). Given the tremendous spatial and temporal variability in toxin concentrations, LHJs are encouraged to factor in the spatial extent of the bloom when deciding if a warning level or closed level advisory is warranted.

Tier II

When recreational guidance values for microcystin, anatoxin-a, cylindrospermopsin and/or saxitoxin are exceeded (Table 1), the LHJ posts a WARNING sign (Figure 6 and Figure 7). The lake is sampled weekly, because toxin levels may be variable, e.g., they may be at their highest during bloom die-offs even though the water looks “normal” or may be significantly lower due to temporary changes in weather such as heavy wind and/or intense rainfall which could redistribute cyanobacteria throughout the lake and throughout the water column with little change in the total number of cyanobacteria cells. This makes assessment of bloom density quite difficult. Therefore, DOH recommends that LHJs not lift advisories unless they check the lake under weather conditions that are conducive to biomass accumulation (relatively calm or a light steady wind and little or no rainfall).

Additional steps can be taken to communicate risk (*i.e.*, press release, notification of veterinarians and fish and wildlife officials) depending on severity of the bloom, time of year, and historical use of the lake (*i.e.*, a highly used access point such as a dog park might warrant greater outreach efforts as compared with a lake not known for any recreational activity). In certain situations, some LHJs have mailed notifications to local lakefront residents after confirmation of cyanobacterial toxicity. Other possible measures that have been used to reach lakefront residents include radio messages or the internet via a list serve or “blast” email.

Tier III

Under certain circumstances, a LHJ may close a lake with unusually high microcystin, anatoxin-a, cylindrospermopsin, or saxitoxin concentrations. At the discretion of the LHJ, a water body can be posted as DANGER—Closed (Figure 6 and Figure 7). Examples include:

- Very dense blooms covering an entire lake
- Confirmed pet illnesses or death
- Reported human illness

The LHJ will post a press release to notify the general public of a lake closure. Also, LHJs follow whatever additional methods of outreach, including those listed under Tier II, that best inform public beach users and lake front residents of the risks from cyanotoxins and how to avoid these risks. Retraction of lake closures is also at the discretion of the LHJ. DOH recommends posting a WARNING sign and following Tier II recommendations after retracting a lake closure until microcystin levels are less than the recreational guidance levels (Figure 2).

## 8. Human Illnesses Associated with Freshwater HABs

Human illness reports following HAB exposure are investigated; however, definitions of suspected, probable, or confirmed human illnesses have changed over time making quantitative reporting problematic. Symptoms following exposure are similar but criteria for illness reporting have changed. At present, the CDC is working on case definitions for national consistency in reporting human illnesses following HAB exposure with the realization that underreporting likely is an issue. Acknowledging these shortcomings, DOH reported 2–4 human illness investigations per year for 2010–2013, with a high of 122 human investigations in 2009, a year with unusually high temperatures during late July–early August.

The risk of illness due to exposure to toxins in freshwater will be reduced through more extensive communication and outreach. To that end, Washington has a database of freshwater toxicity data available for the public to access via the web ([8]; Supplementary Figure 1). Toxicity results can be searched and retrieved by lake, county, water resource inventory area, and toxin with defined concentrations and dates (e.g., Figure 2).

## 9. Future Threats, Needs and Recommendations

Although our understanding of toxic blooms in marine waters and state lakes is improving each year, many questions remain. Below is a list of suggested topics recommended for future work on freshwater and marine HABs in Washington.

- Lake and reservoir HABs in Washington pose a potential new public health threat from exposure via drinking water. In 2014, a 500 household community used untreated drinking water from a lake during a period when anatoxin-a concentrations were low but still above state recreational guidelines; no illnesses were reported. In another case, the drinking water source for Friday Harbor, an island town, had a toxic bloom that resulted in the need to import water for the community. Future efforts will be needed to improve testing in lakes used as drinking water sources and to coordinate with drinking water managers of surface water systems that may develop toxic blooms.
- The additive toxicity of co-occurring blooms in lakes and marine waters must be studied. Further, as microcystin variants become easier to identify and quantify, toxicologists will need to determine actual toxicities to improve upon the current assumption for public health guidance that all toxin variants are equally potent. In the future, our state will adopt national recreational values for freshwater cyanotoxins following EPA guideline development. CDC and the states are collaborating on an enhanced National Outbreak Reporting System that will fill the current gap at the state level for tracking animal and human health illness events.
- The impact of climate change on marine HABs and cyanobacteria is also a subject that needs to be addressed. Cyanobacteria and some marine HABs favor warm temperatures and other environmental conditions such as increased nutrient inputs from land that will be associated with climate change. If long-term climate projections for the Pacific Northwest are correct, rain events will increase, which may influence nutrient runoff from impervious surfaces, particularly as land is developed and regional populations increase.
- Washington has an effective Freshwater Algae Control Program based on passive surveillance, legislatively funded toxicity tests, and established cooperation between state agencies and local health jurisdictions. The state’s 39 counties (35 local health jurisdictions) have a range of staff and resources available for water surveillance and sampling. Therefore, this program, together with the marine biotoxin monitoring program, will require continued and repeated outreach efforts to local health jurisdictions regarding blooms, toxicity testing, and toxicity postings. Thus, periodic seminars and webinars will be needed to ensure all areas of the state are aware of the program and knowledgeable about state-level technical support.
- Outreach efforts on marine and freshwater HABs have met some needs but other educational needs remain unmet. Outreach to veterinary clinics regarding differential diagnoses and distribution of posters for pet owner education has been effective in the state. Annual outreach to the public and to hunters owning dogs will need to continue. More recently, DOH has included outreach to drinking water operators about available toxicity tests, bloom identification, and options for treatment when blooms occur. However, a major outreach and education gap in the state is for physicians who treat those exposed to toxic marine and freshwater blooms.
- Standardized and consistent posting at lakes and shellfish harvesting beaches experiencing toxic blooms is essential for public health protection. Some local health jurisdictions have raised concerns about over-posting, which can lead to the public ignoring CAUTION and WARNING signs, and under-posting, which may not be protective of public health. Since blooms in lakes and marine waters are notoriously patchy, some areas of a lake may be below recreational standards while high toxicity scums in smaller areas remain a health threat. We recommend that managers, local health professionals, and state staff work together to refine outreach and offer additional posting options to reflect more complicated local conditions.
- Recommendations for future work include ongoing collaborative work investigating the link of freshwater toxins with marine bivalve bioaccumulation of toxins. Further investigation of HAB genetics may help explain why some blooms are toxic and others are not. Another recommended effort is to investigate if satellite imagery using smaller pixels can identify lakes with dominant cyanobacteria that are not under current surveillance.

## 10. Overall Summary and Conclusions

The integration of phytoplankton monitoring into regulatory programs to ensure shellfish safety has been promoted by European countries for many years and should be encouraged throughout the U.S., in particular in those states, such as Alaska, where regulatory monitoring of vast coastlines is challenging [102]. In some regions of the U.S., including Florida, cell counts of harmful algae are used together with satellite imagery and automated environmental observations to provide early warning of the development and movement of *Karenia brevis* blooms in the Gulf of Mexico [103]. Currently, each European state monitors marine HAB species in addition to toxins in shellfish along the Atlantic coastline. These data are interpreted and incorporated by each national monitoring program into national forecast bulletins that were developed during the program, Applied Simulations and Integrated Modeling for the Understanding of Toxic and Harmful Algal Blooms (ASIMUTH) project, as a demonstration of a downstream service [104].

For freshwater HABs, each state has developed a unique approach for monitoring and regulating toxic blooms. The most extensive effort is in Florida, where the Florida Department of Health (FDOH) has developed the Harmful Algal Bloom Online Tracking Module, which allows public health professionals and environmental scientists/managers to collaborate on cyanobacteria bloom reporting through a secure web-based data management system, hosted in *Caspio.* There are currently 86 users from 18 different organizations utilizing the system. Other examples of states integrating monitoring into regulatory programs include Oregon’s collaboration with the State Drinking Water Program and an effective emphasis on education and outreach; Massachusett’s collection of bloom data to serve as guidance for local health officials; New York’s collaboration with the Citizen Statewide Lake Assessment Program; Wisconsin’s interactive website for incident reporting by citizens and local health departments and strong partnerships with WI Department of Natural Resources and poison control centers; Maryland, Virginia, and South Carolina’s integration of freshwater and marine HAB monitoring and tracking of blooms; and Iowa’s effective partnership between health and natural resource departments. Such programs provide an effective interface between formalized state and federal programs while observations by trained citizen volunteers offer the best possible early warning systems for surveillance of known HABs as well as for the reporting and diagnosis of unusual events that may impact the future health of oceans, lakes and humans.

The vision for the future includes interfacing current monitoring and management programs with efforts in basic research and model development to develop forecasting systems for marine and freshwater HABs. Early warning networks will monitor changes in the abundance and location of toxic blooms using an integrated suite of sensors on satellites and stationary sensor platforms that together can measure ocean water properties including temperature, current speed and direction, chlorophyll, cell species and abundance, and toxins. Data will be telemetered and incorporated with real-time shore-based monitoring. An example of a remote sensing technology is the automated molecular detection and quantification of cells and toxins, using the environmental sample platform (ESP; e.g., [105,106,107,108]), which recently has been funded for deployment in the Pacific Northwest as part of the Integrated Ocean Observing System [109]. Rapidly-accessed data from remote platforms will be used to calibrate and fine-tune physical and biological models and HAB forecasts. One such forecasting bulletin for the Washington State coast is in its pilot stage [110]. These models and forecasts will allow shellfish managers and early warning programs to take preventive actions (such as increasing monitoring efforts, closing targeted shellfish beds, and warning at-risk communities) to safeguard public health, local economies and fisheries. In addition, some proactive management will be facilitated, e.g., early opening of the shellfish harvesting seasons or early posting of toxin threats to recreational users of lakes. A combination of technologies, from volunteer-based phytoplankton monitoring programs, to state and federal regulatory analysis of toxins in shellfish and drinking water, to the newest remote sensing technologies, will provide the most comprehensive system for the protection of public health from documented marine and freshwater HABs as well as new and emerging biotoxins in Washington State.

## Supplementary Materials

Supplementary materials can be accessed at: http://www.mdpi.com/2072-6651/7/4/1206/s1.
