# Supplementary material for: Integrative Monitoring of Marine and Freshwater Harmful Algae in Washington State for Public Health Protection
Source: Toxins (Basel). 2015 Apr 9;7(4):1206–34. doi: 10.3390/toxins7041206 (PMC4417964; doi:10.3390/toxins7041206)
Supplement: Supplementary file 1 [file toxins-07-01206-s001.pdf]

Supplementary Information

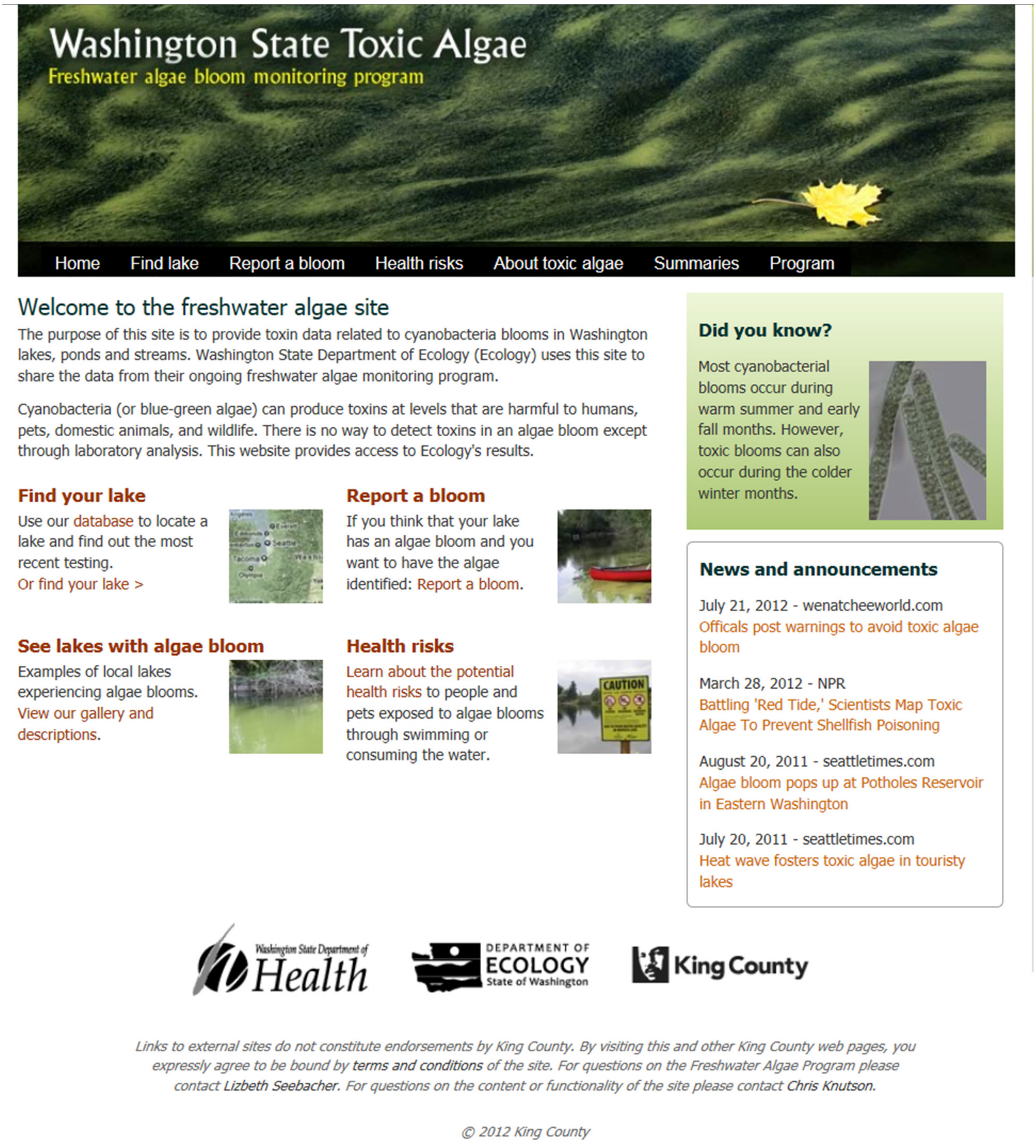

Figure S1. Washington State Toxic Algae website home page [S1].

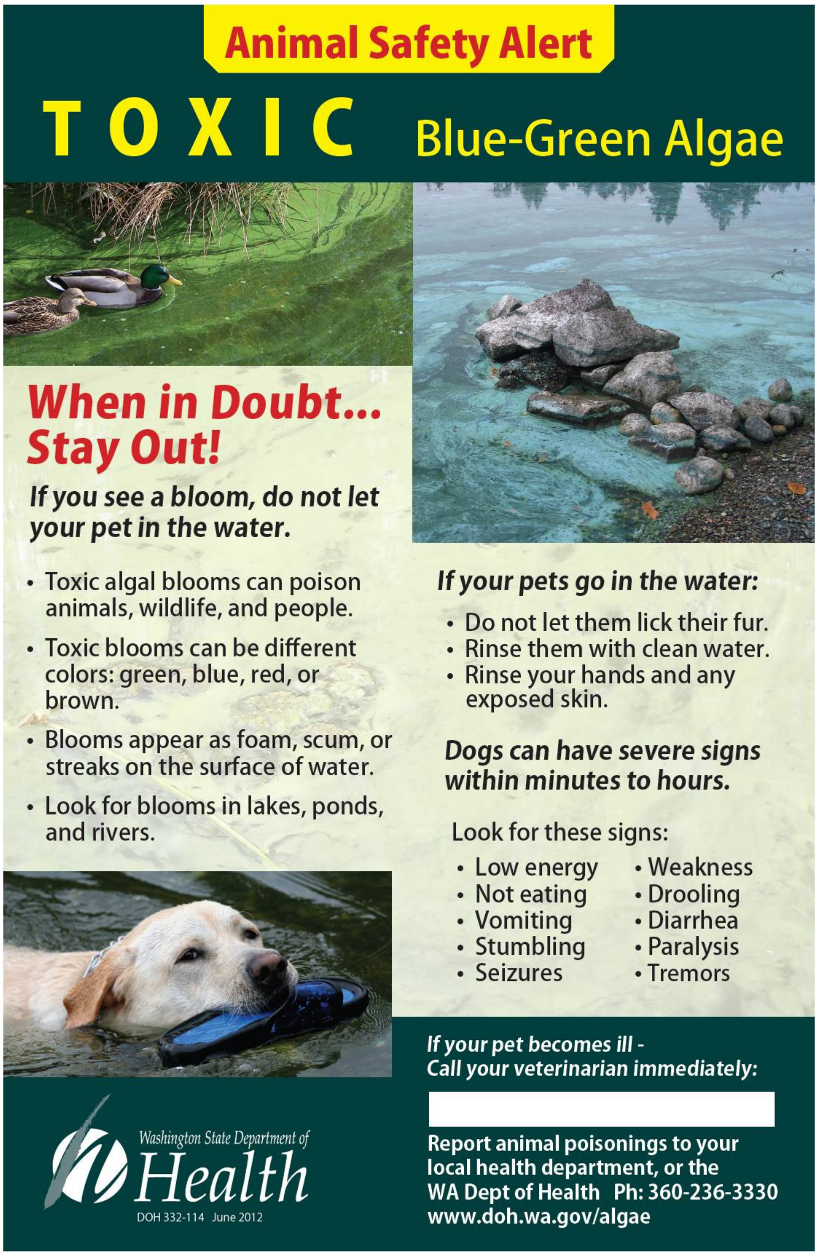

Figure S2. Animal safety alert poster.

Reference

S1. Washington State Toxic Algae, Freshwater Algae Bloom Monitoring Program. Available online: http://www.NWToxicalgae.org (accessed on 9 February 2015).
